# Deep Neural Network and Monte Carlo Tree Search applied to Fluid-Structure Topology Optimization

**DOI:** 10.1038/s41598-019-51111-1

**Published:** 2019-11-04

**Authors:** Audrey Gaymann, Francesco Montomoli

**Affiliations:** Uncertainty Quantification Laboratory, Aeronautical Engineering Department, Imperial College London, London, SW7 2AZ UK

**Keywords:** Aerospace engineering, Mathematics and computing

## Abstract

This paper shows the application of Deep Neural Network algorithms for Fluid-Structure Topology Optimization. The strategy offered is a new concept which can be added to the current process used to study Topology Optimization with Cellular Automata, Adjoint and Level-Set methods. The design space is described by a computational grid where every cell can be in two states: fluid or solid. The system does not require human intervention and learns through an algorithm based on Deep Neural Network and Monte Carlo Tree Search. In this work the objective function for the optimization is an incompressible fluid solver but the overall optimization process is independent from the solver. The test case used is a standard duct with back facing step where the optimizer aims at minimizing the pressure losses between inlet and outlet. The results obtained with the proposed approach are compared to the solution via a classical adjoint topology optimization code.

## Introduction

Topology optimization designates a mathematical approach used to optimize design geometries considering a set of working conditions, constraints and goals. The final designs meet the requirements given based on the space available and offers the optimum solution to the given problem. First introduced in the 80 s^1,2^, it quickly gained traction in the context of structural optimization and with the prospect of revolutionized manufacturing methods brought by Additive Manufacturing (AM). However the specific domain of Fluid-Structure Topology Optimization (FSTO) remains under development.

The domain of FSTO covers the development of engineering designs in the context of structures in contact with a fluid. The structure design is optimized according to the fluid path which drives the solution and performance. The objective of a FSTO is to build for example channels with low pressure losses and high heat transfer^3–5^.

Among the different TO mathematical approaches developed, two methods have stand out and are currently attracting research interest in the domain of FSTO: the Level-Set Method (LSM)^6^ and the Adjoint based method (ABM)^3^.

The LSM relies on a starting design whose shape is free to be updated along its contour. From there on, the configuration is slightly modified following an optimization process^6^. LSMs have been widely used for structural optimization as they give good flexibility on handling topological changes, as well as fidelity of boundary representation^7–9^. The main drawback is the fact that the geometrical overall topology must be preserved and the high computational cost, due to the number of numerical simulations (FEM/CFD) to estimate the optimal performance.

To overcome these limitations, adjoint based methods are becoming more popular. Adjoint formulation relies on the definition of the derivatives of the dependence of a specific performance indicator (such as efficiency) to a geometrical variation^3^. The advantage of such formulation is the ability to deal with a very high number of design parameters. In recent formulations, adjoint optimization has been coupled with sedimentation algorithms, to allow the optimizer to generate solid zones in the domain by changing the permeability of each cell. The main drawback of such method is the fact that it is an inherently local optimal strategy, some time the code uses porous cells and the flow instabilities can alter the convergence of the code. At the moment, Direct Navier Stokes simulations cannot be used for adjoint optimization.

Applications using the methods described have been widely documented in the literature for problems related to structural topology optimization. However, applications for fluid-structural problems are scarcer with little work done on three dimensions, due to the complexity of modeling flows behaviour. Some examples can be found by the recent work of^10^, who minimized the pressure losses. Other examples using LSM include^11,12^ while an adjoint formulation has been used by^3–5^.

Another concept for TO which did not get the same momentum as the previous methods described is the concept of Cellular Automata (CA), where the concept mimics the behaviours and patterns observable in nature. The principle relies on the same base as the adjoint with a grid formed of cells^13^. However the cells here have only two states available (binary/discrete state): either solid or liquid. One cell state is a function of its local neighbours and the system are stacked based on a rule set to obtain designs. CA takes its origin in a TO category referred to as non-gradient topology optimization (NGTO), as opposed to the gradient topology optimization (GTO), as seen with LSM and ABM. Though critics have been formed concerning the utilization of NGTO due to poor representation of the physical problems or due to the cost of the simulations combined with the difficulty it has on finer scale^14^ in structural TO, they still offer an alternative where GTO fails. Especially in the domain of FSTO where the rendering of turbulence models and physics of the flows becomes challenging for gradient-based method that are particularly sensible to discontinuities and instabilities resulting from the flow behavior.

NGTO, or generally heuristic-based approaches, are not particularly common in the field of FSTO. They have been developed and used in the field of structural TO for the interest that they do not rely on a gradient and offer a simpler implementation. A direct consequence of it is the implication that they can be used for any type of analysis. Methods based on genetic algorithm^15^ are documented for example for both structural^16^ and fluid optimization^17^.

As stated, the GTO methods described have their pros and cons, though they do not offer a universal method which can be used in all circumstances, especially in the domain of FSTO. Consequently an alternative method for TO, based on NGTO, which could offer a higher flexibility than the current methods available would be of high interest. With the rise of Machine Learning (ML) and its large range of application, the utilization of methods based on Deep Neural Network (DNN) could offer a good alternative and this is investigated in this paper.

TO, at its core, is essentially a design which is represented as an image for 2D cases. Consequently, the ML algorithms developed for images such as as pattern recognition network and image enhancement methods become viable methods for TO applications. These design images have also an interesting property of being binary (solid or liquid), and thus bear similarities to games such as Go games and Chess, where players use black and white pieces.

Following this resemblance, it came naturally to consider one of the most high profile ML algorithm developed the past few years: AlphaGo. In 2015, AlphaGo made history by being the first computer Go program which managed to beat a human professional Go player without handicaps on a full-sized 19 × 19 board. AlphaGo algorithm went on in the next two years to beat the world champion. What is remarkable about this system was the victory of the machine over the human when the number of possibilities were until now too high to predict for a computer. The ML algorithm of AlphaGo relies on Monte Carlo Tree Search (MCTS) and Deep Neural Network (DNN), enabling it to learn from a zero state, to play against itself and to predict the best move at a state t in time without the need to map all the possibilities^18,19^.

An interesting point to note is the similarity between cellular automata, or more generally NGTO, applied to TO and a Go Game. They both rely on a grid with two binary value possible inside and even if the rules are different, the set-up remains similar. The main interest in such method is the possibility of developing a TO system which can work with any CFD software and the possibility to use Direct Navier Stokes (DNS) simulations to train the dataset and optimize the design space.

In this paper, a framework similar to AlphaGo is used to build a TO solver based on machine learning. The framework involves a Monte Carlo Tree Search and a CFD solver to compute the global performance. The test case used is a step geometry for which the optimum solution, obtained with other methods, is well known and it will be used as reference. Three grids are tested: a grid of ${{\bf{G}}}_{1}\ni [2\times 5]$, a grid of ${{\bf{G}}}_{2}\ni [4\times 10]$ and a grid of ${{\bf{G}}}_{3}\ni [8\times 20]$. The framework is completely general and any test case can be modelled. The method can be applied both for structural topology optimization and FSTO. The rules governing the game of the Topology Optimization can be updated depending on the need: for example a rule stating that the solid state can only occupy a given percentage of the total volume can be added to constrain the result.

## Test Cases and Results

### System information

Three test cases are tested to validate the method. The optimum configuration found by the best network is used and compared to a reference optimum obtain by a standard adjoint method.

The objective of the optimization is to decrease the pressure difference between inlet and outlet, noted as Δ*P*. The winner of the game is defined as having the lowest Δ*P* between Player 1 (P1) and Player 2 (P2).

**Figure 1:**
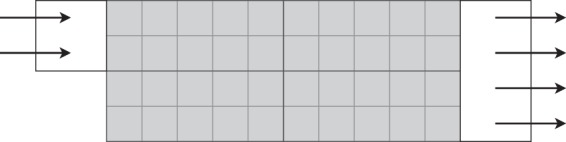
Design Space of the step geometry used as test case. The inlet is represented on the left, the outlet on the right.

Note that the grid, or mesh, will be referenced as design space. The test case studied is a step geometry as seen in Fig. 1. The flow enters from the left hand side and exit the geometry on the right hand side, as indicated by the arrows. The geometry dimensions remain constant and the number of cells present in the grid is increased from 10 to 40 to 160 to build the three test cases, as described in Table 1. Each cell can take either a fluid or solid state during the optimization. One grid of the same size is given to each player. For **G**_1_, the number of possibilities available are 2^10^ while for **G**_2_ it is 2^40^ and **G**_3_ it is 2^160^.

**Table 1:** Test cases grid size.

Grid (G)	H	L	W	Number of configurations (max)
1	2	5	1	2^10^
2	4	10	1	2^40^
3	8	20	1	2^160^

Note that the height H, the length L and the width W are counted in number of cells. The dimensions and the Reynolds number remain constant from **G**_1_ to **G**_3_.

**Figure 2:**
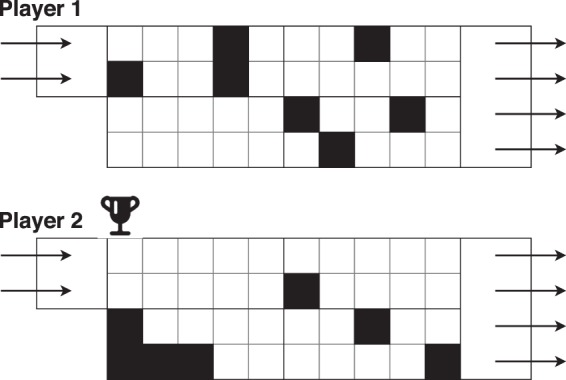
Game situation. Two networks compete to obtain the smallest pressure difference between the inlet and outlet. White cells are in a fluid state while black cells are solid.

Player 1 algorithm competes with player 2 algorithm in order to obtain the best geometry, as seen in Fig. 2. The algorithm yielding the best answer will be decided as optimum configuration.

**Figure 3:**
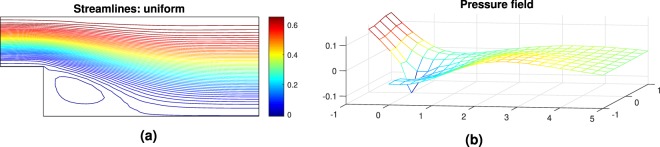
Step geometry velocity streamlines (**a**) and pressure field (**b**) for the design space configuration. Note the re-circulation area at the bottom left corner.

Note that in the step geometry, a re-circulation area is present and it is responsible for a loss of pressure globally as seen in Fig. 3. The penalization of such area is expected though the form it is going to take depends on the quality of the mesh. The higher the number of cells the more accurate the profile of the optimum geometry becomes. However, the interest here is to find a logical pattern in the update of the geometry. If a correlation is found between new design and lower pressure losses as given by the CFD solver, then the machine learning applicability for FSTO will be proven.

### Calibration and training

The system learns from a blank state and through numerous iterations build random games which can be used for the calibration of the networks. The method was used on the three test cases. Note that it is possible to control the MCTS expansion to limit the number of possibilities using engineering common sense and start the system with a calibrated network relying on specific random data to speed up the process, especially in the test case of **G**_3_.

Indeed, for a grid of 20 × 8, a system with more than 20% solid state cells randomly created builds blocked structure at a high rate. A blocked structure impedes the flow to circulate. As the goal is here to decrease pressure losses, a fully blocked system represents very little interest. Consequently random configurations were created by limiting the amount of solid cells inside the total volume for **G**_3_ on the first games batch, as described in Table 2.

**Table 2:** Random data created for the first calibration of the Network for **G**_3_.

Number of configurations	100	200	300	200
Solid state (% of total volume)	2.5	5	10	20

Note that the only constraint is the number of solid cells. No constraints was given about their position inside the grid.

These 800 random configurations are used to calibrate the initial deep neural network. For all the systems, the configurations are ranked based on the pressure difference. As the rule of the game is to obtain the minimum pressure difference between inlet and outlet, the *n*_**G**_ best games according to this rule are selected to train the network. The value of *n*_**G**_ is dependent on the network architecture (number of layers and filters), but also on the test cases studied.

To sum up, the first batch of configurations is added to the initial dataset **D**_*init*_. As the games are played, all the configurations are added to the dataset **D**_*Tot*_, while the best configurations are used to build a new dataset **D**_*Int*_ to calibrate the DNN. Best is defined as minimum Δ*P*, with identical structures removed. Non physical solutions, such as fully blocked structures impeding the flow to come from inlet to outlet, or reverse flows, are heavily penalized in order to remove them from a possible selection. Once a game is finished, the winner is defined as the one having the lowest pressure difference and it is confirmed by the modified IFISS. The values obtained for the pressure difference are recorded to build the future training set.

The 3 test cases are done with fixed boundary conditions: the Reynolds number is constant as well as the spatial dimensions. Only the mesh is changed. Note that any CFD solver can be used to compute the difference of pressure of the new configuration. Slight variations are expected in the difference of pressure across the test cases for the Δ*P*_*def*_. It represents the difference of pressure in the empty design space and depending on the quality of the mesh, accuracy will change the expected value. The same comment is made on the optimum solution Δ*P*_*opti*_, values will varies due to the mesh quality. As the main goal is to identify the capacity of the algorithm to find an an optimum configurations with the minimum pressure difference, the accuracy of Δ*P* is irrelevant from the moment the same solver is used to compute all the data.

The algorithm is let to play until the verification game for one of the player yields a configuration where

1 $$\Delta {P}_{def}\ge \Delta {P}_{{P}_{i}}$$

The value Δ*P*_*def*_ represents the difference of pressure in the design space when no solid update has taken place. The value Δ${P}_{{P}_{i}}$ represents the difference of pressure associated to the configuration of the Player *i*. Once the statement held is true, each validation games is controlled. If three consecutive game verification yield the same optimum solution for a given player, the algorithm is stopped and the best geometry is decided on player yielding the smallest repetitive Δ*P*.

### Test case 1

The first test case is the smallest grid **G**_1_. The main interest of working on such basic grid is to confirm on a small scale that the system will behave as expected while using little computing resources as the number of configurations available are very limited, 1024 to be precise. Moreover, it will give good ideas about properties which might influenced the ML algorithm.

The system was let to play randomly until 50 configurations were obtained. From their analysis it could be deduced that some configurations could potentially greatly influence the calibration and testing of the weights and biases value. From there on, a skewed dataset would be defined as a dataset with very low value of pressure losses and very high value with almost no values in between the two extremes. Fitting a ML algorithm on such extreme values would slow down the convergence of the system as it would steer the FSTO in a specific direction. This specific behavior explain why it was decided to keep configurations with low pressure losses of the same order to calibrate and test the system.

**Figure 4:**
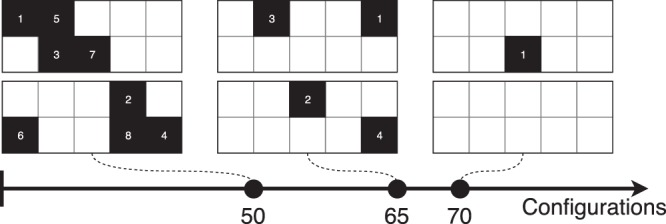
FSTO update by the DNN as the number of games increases to feed in the network. For each two grid set, Player 1 is at the top, Player 2 at the bottom.

The amount of configuration being low, the 90% was dropped to 80% for splitting data between the validation and testing set. The evolution of the configurations as the exploration goes on is represented in Fig. 4. As the number of configurations are increased, the system slowly decreased the number of solid cells. The number represent the order of the update after each turn.

**Figure 5:**
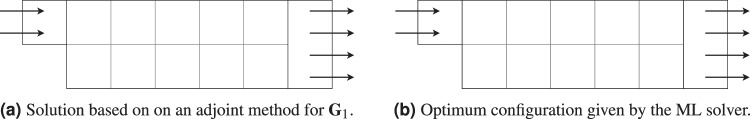
Comparison of the optimum design between adjoint and ML solver for **G**_1_.

The optimum solution with an adjoint system is found to be an empty grid as seen in Fig. 5a. During the optimization process the same result was obtained by the algorithm, Fig. 5b.

**Figure 6:**
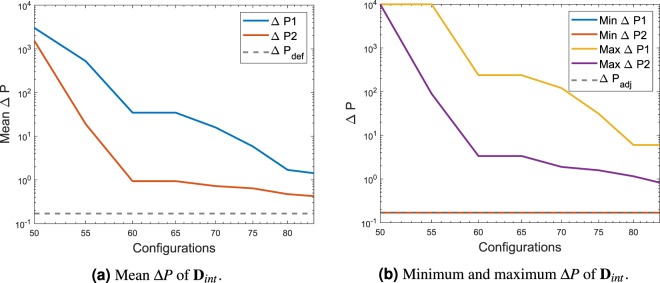
Mean Δ*P* and minimum and maximum Δ*P* of the data extracted **D**_*int*_ for the DNN calibration and testing. The test case represented is **G**_1_.

The mean pressure of **D**_*int*_ and the minimum and maximum values are sum up inside the Fig. 6a,b. As expected the Δ*P* values present in the data set used for the calibration of the DNN keeps decreasing as the MCTS explores the different possibilities. The fact that the optimum solution was already found is explained by the fact that the empty configuration being the default first configuration was tested automatically by the solver at the start of the iteration process.

**Figure 7:**
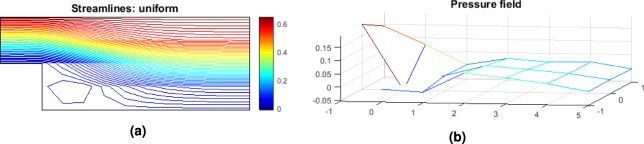
Step geometry velocity streamlines (**a**) and pressure field (**b**) for the optimum design configuration.

The optimum geometry velocity streamlines and pressure field obtained for the optimum configuration are represented Fig. 7.

**Figure 8:**
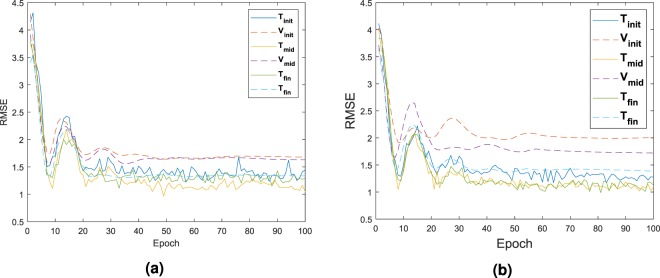
RMSE of Player 1 in (**a**) and Player 2 in (**b**) for the validation and testing dataset, applied with the test case **G**_1_.

The validation dataset **V**_*i*_ and testing dataset **T**_*i*_ which includes the input and output used during the calibration of the DNN had their respective RMSE value recorded as seen in Fig. 8a,b. The first calibration is represented with the subscript’init’ for initialization. The calibration at half way through the process is represented with the subscript’int’ for intermediate. The final calibration is represented with the subscript’fin’ for final. As it can be noticed the best fit is happening on the last calibration of the network, with the least difference between the RMSE of the testing and validation set. This is also due that at this moment, configurations fed inside the system are closely related and all extreme cases which might skew the data have been removed.

### Test case 2

**Figure 9:**
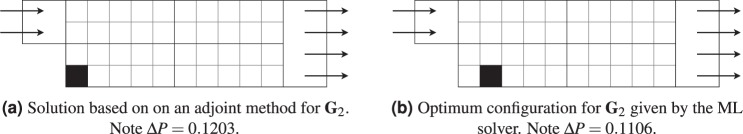
Comparison of the optimum design between adjoint and ML solver for **G**_2_.

The grid **G**_2_ already offers a more challenging system. The adjoint solution is given in Fig. 9a. As expected, the re-circulation area is being penalized. However, the optimum answer found by the ML solver is slightly different as seen in Fig. 9b. The pressure difference was confirmed by a CFD solver and it is found that the optimum in this case is given by the ML solver.

**Figure 10:**
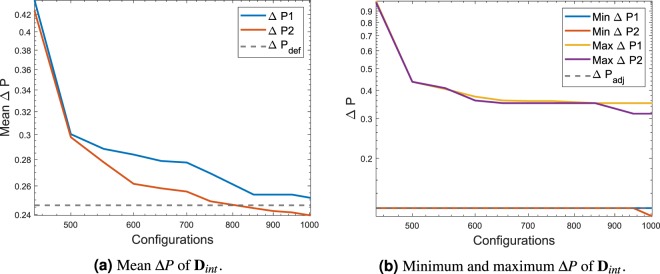
Mean Δ*P* and minimum and maximum Δ*P* of the data extracted **D**_*int*_ for the DNN calibration and testing. The test case represented is **G**_2_.

Figure 10a shows the convergence of the mean pressure difference in the data used for the calibration and testing of the DNN. As the number of configurations increases, the mean value decreases, as expected for a FSTO problem where pressure difference needs to be decreased. The reference value Δ${P}_{def}$ is the difference of pressure of the design space in the absence of solid material. Figure 10b shows the minimum value and the maximum value present in each dataset and it is compared to an adjoint solution difference of pressure. Data shown starts at the configurations 400 as at lower values the configurations present in the algorithm had very high Δ*P* values, mostly due to blocked structures.

As observed in the previous case, the trend goes down as the number of configuration increases which confirms the behaviour of the algorithm in searching for a minimum Δ*P*.

**Figure 11:**
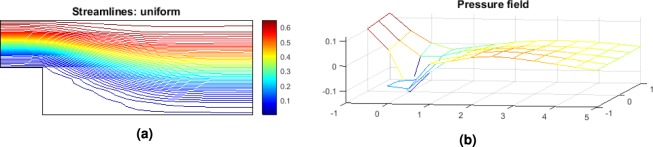
Step geometry velocity streamlines (**a**) and pressure field (**b**) for the optimum design configuration.

The optimum geometry velocity streamlines and pressure field obtained for the optimum configuration are represented Fig. 11.

**Figure 12:**
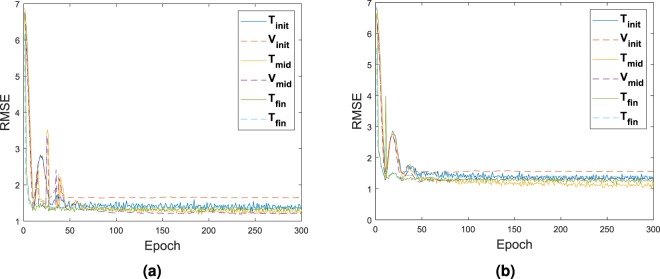
RMSE of Player 1 in (**a**) and Player 2 in (**b**) for the validation and testing dataset, applied with the test case **G**_2_.

The comparison of the training based on the RMSE is sum up in Fig. 12a,b. The first training as well as the mid point training and the last training are represented only. This time, due to the larger amount of data, it was possible to select the data such that skewed value would not impact the process.

**Figure 13:**
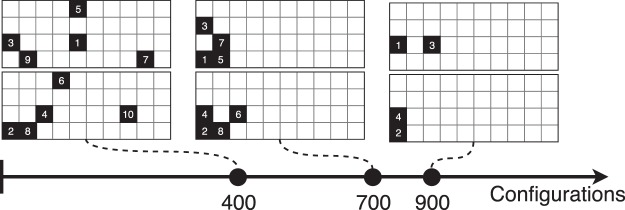
FSTO update by the DNN as the number of games increases to feed in the network. For each two grid set, Player 1 is at the top, Player 2 at the bottom.

An evolution of the DNN game state is represented in Fig. 13. As the number of configurations increases, the player algorithm learns how to update the system in order to decrease the pressure loss. It can be noticed that the cells considered as solid, if at the beginning they spam the design space, they slowly converge to the area of interest which is the re-circulation area.

### Test case 3

The third test case offers a different optimum configuration from the adjoint for both P1 and P2, however the pressure difference remains the same between the three different configurations.

**Figure 14:**
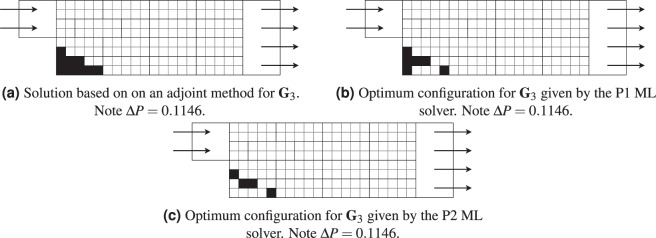
Comparison of the optimum design between adjoint and ML solver for **G**_3_.

Concerning the difference between P1 in Fig. 14b and the adjoint in Fig. 14a, as stated before, the mesh tested is extremely coarse. The boundary layer won’t be accurately modelled by the solver, implying discrepancies in the results with what a fine mesh would have obtained. However the interest remain the acquisition of a design with a minimum pressure difference between inflow and outflow and the algorithm still obtained an optimum configuration as both adjoint and the ML algorithm obtained the same pressure difference with the solver.

As for the difference between P1 in Fig. 14b and P2 in Fig. 14c, note that both designs are similar with the exception of two cells. These specific cells are below solid cells that are forming a wall, implying that the flow does not reach them. The end result is that the design of P1 and P2 are actually the same. This is of high interest as rules can be added to take care of such designs and speed up the convergence. Such a rule would in practice solidify any cells surrounded by solid cells as no flow is circulating inside them and they don’t actively participate to a meaningful convergence. Moreover, it can be seen that the ML algorithm is actually trying to win with the lowest amount of turns, implying the utilization of only the necessary cells to achieve this goal.

**Figure 15:**
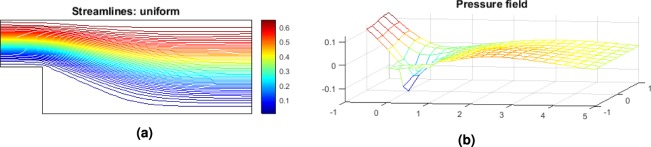
Step geometry velocity streamlines (**a**) and pressure field (**b**) for the optimum design configuration.

The optimum geometry velocity streamlines and pressure field obtained for the optimum configuration of P2 are represented Fig. 15.

**Figure 16:**
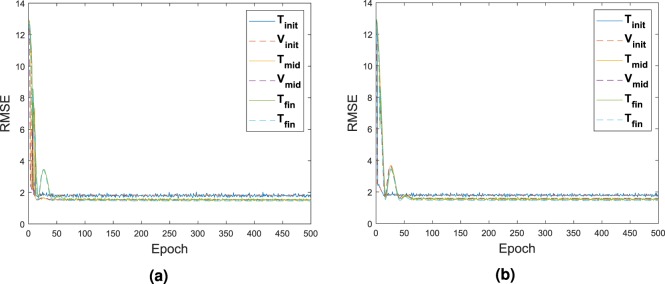
RMSE of Player 1 in (**a**) and Player 2 in (**b**) for the validation and testing dataset, applied with the test case **G**_3_.

The comparison of the training based on the RMSE is sum up in Fig. 16a,b. Again, as stated before, the first training as well as the mid training and the last training are represented only. The selection only takes the best solution. Very little difference is observed on the error.

**Figure 17:**
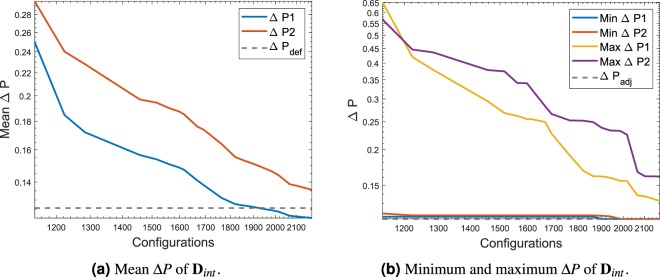
Mean Δ*P* and minimum and maximum Δ*P* of the data extracted **D**_*int*_ for the DNN calibration and testing. The test case represented is **G**_3_.

Figure 17a shows the convergence of the mean pressure difference in the data used for the calibration and testing of the DNN. As seen in the two previous test cases the mean value keeps decreasing as the number of games are played. Figure 17b shows the minimum value and the maximum value present in each dataset and it is compared to an adjoint solution difference of pressure. As observed in the previous cases, the trend goes down as the number of configuration increases which confirms the behaviour of the algorithm in searching for a minimum Δ*P*.

**Figure 18:**
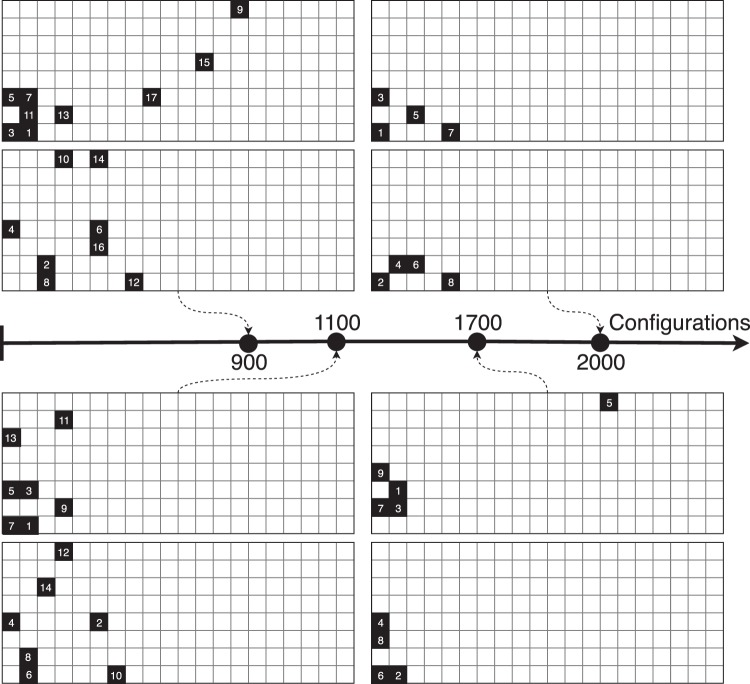
FSTO update by the DNN as the number of games increases to feed in the network. For each two grid set, Player 1 is at the top, Player 2 at the bottom.

To finish, the evolution of the configurations as the algorithm builds its dataset and plays against itself is represented in Fig. 18. The re-circulation area is again targeted by the solver as an area to penalize in order for victory to happen.

Note that the final optimum configuration can also be compared to the configurations already visited. There is a possibility for the solver to find out relatively early an optimum configuration by itself while visiting the Monte Carlo Tree. The final optimum yielded by the ML algorithm can be validated by a quick search among all the positions already visited.

## Conclusions

The work presented shows that it is possible to use a Machine Learning formulation for fluid-structure topology optimization. The three test cases yielded the optimum design for the given design space, and the difference of pressure was validated by the CFD solver. The approach presented has the advantage of the possibility to use any high order formulation to estimate the objective function (in this case pressure drop).

An additional advantage is its universality: it opens the possibility of using this algorithm for any kind of topology optimization problem, independently of being structural based or fluid-structural based. The system only requires to be connected to a validation software, in charge of confirming the expected value of the objective function. This is a direct consequence of the nature of the algorithm which does not rely on the calculation of the gradient/sensitivity. The method is general and may be applied to other fields of TO like structural optimization. The goal in this case would be to maximize the stiffness while constraining the total volume of the structure^20^ but the approach presented is still applicable. In such case the total volume can be limited using the MCTS exploration with a rule applied on the number of moves available to each player while the stiffness can be calculated by a Finite Element Method (FEM) solver on the final design. The solver would be able to explore the space of solution of the test problem and convergence to an optimum for the structural TO test case.

Moreover, the proposed method overcame some of the limits of adjoint based fluid TO. The solver used in CFD problems, such as RANS solvers or the turbulence models used, are not anymore a constraint on the system thanks to the global search of the performance value. These models won’t be a source of instabilities as the geometry would be converged for the performance global value. An interesting by-product of the current method is the exploration of the space of the solution. As the dataset **D**_*Tot*_ records all the configurations explored as well as their performance value, the solver has a partial coverage of the space of solution for a given design space. Consequently, observing different local optima and potentially getting the global optimum is an interesting feature of the implementation. The solver output can be at any moment challenged by what is available inside the dataset.

The cons of the method developed are based on the amount of CFD simulations required to build an optimum design. Each new update of the game state needs to have its Δ*P*, or in a general way its objective function, calculated if it corresponds to a new configuration. However, note that the system is flexible enough to have rules added to the system to control and limit the exploration of the MCTS. This would directly constrain the search inside the space of the solution, leading to a more efficient exploration of the optima.

This new method shows promises as an alternative to current topology optimization methods. Further possible work on the subject would be including an image refinement algorithm which could enable a speed up of the method for finer meshes. Indeed, running a TO optimization problem on a coarse mesh could then be extrapolated to finer mesh with image enhancement machine learning algorithm.

## Methods

This section will go in details about the implementation of the Fluid-Structure TO. The algorithm developed by Deepmind for AlphaGo is used as reference to develop the same pattern recognition system applied to TO. The optimization is self-play and without human inputs. There are three main components to the implementation: the fluid solver, the Monte Carlo Tree Search and the Deep Neural Network.

### Input and output

The definition of input and output of the network will influence the optimization of the system. The MCTS relies on the DNN for its expansion and the DNN is calibrated on the values given as input and output. Note that the system relies on 2 similar DNN algorithms, each attributed to a player and calibrated with its data. The algorithms compete against each other in order to obtain the best geometry based on a defined target such as pressure losses.

**Figure 19:**
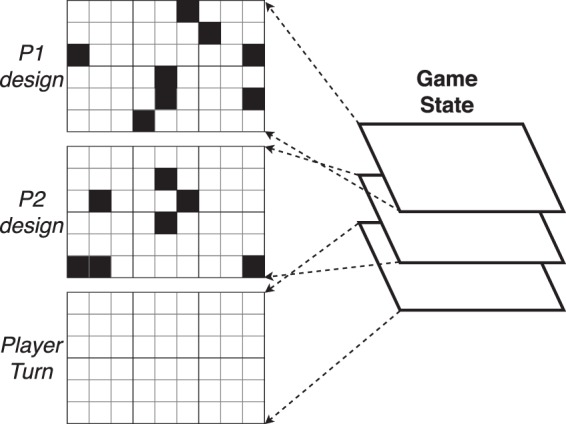
INPUT of the DNN. The Game state is a stack of the design built by the player P1, the design of player P2 and the player turn grid.

The input of the DNN algorithm is defined as the Game State, as seen in Fig. 19. It is a stack of three binary images. The first image is the state of the design for player P1. The second image is the design given by player P2. The last image is either a binary image where all the pixels have a value of 0 when it is P2 turn, or a value of 1 when it is P1 turn. Every time a player updates its grid, the stack is updated and fed in the DNN.

**Figure 20:**
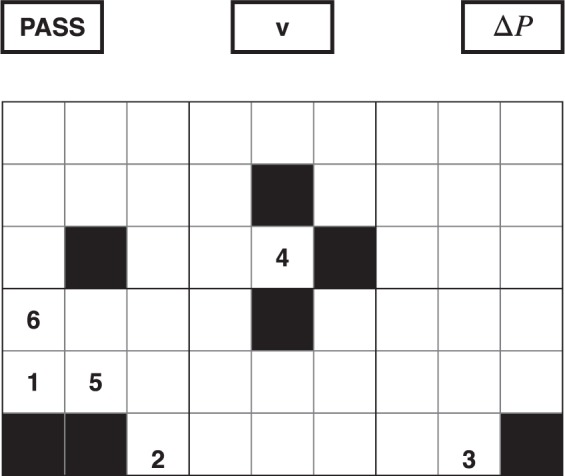
OUTPUT of the DNN. It gives a probability value for the next moves (here ranked by priority), for the player to undertake a pass action, to win and an estimation of the difference of pressure for the current design.

The output of the DNN is shown in Fig. 20. It gives access to four different data: a grid with the probability *p* of each move given, a Pass value, a victory probability value *v* and the difference of pressure Δ*P*. The first output has the same size as the grid and it gives for each position the probability of it being played. The value *p* is needed in the MCTS. The second output is the PASS value; it is a probability estimating if the player should pass or continue playing. A PASS move implies the algorithm estimates it has the best design available in the grid. The third value is a probability of victory *v*, needed in the MCTS. The fourth output is the pressure difference. The algorithm tries to predict the pressure difference of the current configuration played inside the Game State.

The decision of working on binary images stems from the similitude between a binary image divided in pixel, with a Goban divided in a grid, with a TO mesh study divided in cells.

On a binary image, the pixels are either of value 1 or 0 to represent the image. On the Goban, the players play with white and black stones. A TO structure is represented by cells which are either fluid or solid. This high similitude explains the fit of the algorithm between the test problems.

Note that theoretically the solver works for 2D and 3D meshes with TO problem. Extending the 2D grid to a 3D grid would not be a problem if the third dimension is considered as a stack of 2D slices. However, note that the number of combination increases following the rule ${2}^{{N}_{x}\times {N}_{y}\times {N}_{z}}$ where *N*_*i*_ represents the number of cells along the **i** spatial dimension. The value 2 is due to the binary state of the cell: the value 0 implies the cell is in a fluid state while the value 1 is associated to a fluid state.

### Deep neural network

The architecture used is a combination of a convolution layer and residual layers as described in the previous section. The input dimension is a stack of ${N}_{x}\times {N}_{y}\times 3$, dependent on the test case. The DNN is divided in three consecutive blocks.

The first block architecture is based on a convolution layer and follows the pattern:

1. ${n}_{C{f}_{i}}$ convolution filters of 3 × 3 with stride 1
2. Batch Normalization
3. Rectifier non-linearity

The second block adds a total of ${N}_{R{l}_{i}}$ residual layers, with a residual layer defined as:

1. ${n}_{C{f}_{i}}$ Convolution filters of 3 × 3 with stride 1
2. Batch Normalization
3. Rectifier Non-Linearity
4. ${n}_{C{f}_{i}}$ Convolution filters of 3 × 3 with stride 1
5. Batch Normalization
6. Skip Connection
7. Rectifier Non-Linearity

The third block of the DNN gives access to the policy and value head (output) of the DNN. The architecture pattern is as follow for the value head:

1. 2 Convolution filters of 1 × 1 with stride 1
2. Batch Normalization
3. Rectifier Non-Linearity
4. Fully Connected Layer
5. Rectifier Non-Linearity
6. Fully connected Layer
7. Tanh Non-Linearity

For the policy head:

1. 2 Convolution filter of 1 × 1 with stride 1
2. Batch Normalization
3. Rectifier Non-Linearity
4. Fully connected Layer

**Figure 21:**
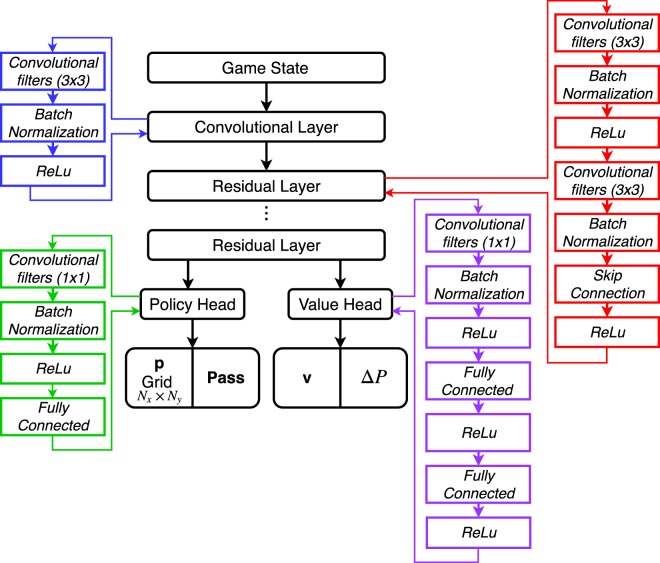
Algorithm of the DNN architecture.

Figure 21 sums up the overall architecture of the DNN. The variable ${n}_{C{f}_{i}}$ and ${N}_{R{l}_{i}}$ values are dependent on the Grid size. The larger the grid, the larger the amount of data required. Consequently, having the same architecture from one grid to another might lead to an overfitting or an underfitting problem.

The value head predicts the winner and give access to the value of the state of the current player *v*, but also the prediction of difference of pressure Δ*P*. The policy head gives access to the move probabilities *P* for the grid coordination and the pass value.

The DNN is trained on a selection of games considered as the best available among all the games. To build a training and validating set, 90% of the best games are selected randomly for the training set and 10% are used for the validating set.

The Loss function used to optimize the network weights rely on a Mean-squared error (MSE) and L2 regularization (as there is a possibility of over-fitting). As all games and results are recorded, it is possible to confirm the validity of the final solution by accessing the data-set built.

### Monte carlo tree search

The Monte Carlo Tree Search MCTS is used to map the possible future moves from both player. Mapping all the different possibilities available inside the design space becomes very quickly intractable with the size of the mesh and consequently the system needs to predict what kind of moves or patterns lead to a win inside the system. The decision of the move would be a direct consequence of pattern recognition.

The Game State *s* is the input of the tree. From there, nodes are created to predict the opponent moves and the probability associated to each moves. The node *n*, representing a possible action *a* in the design space, stores four statistical variables:

2 $$[N(s,a);W(s,a);Q(s,a);P(s,a)]$$

The first variable *N* represents the number of times the nodes has been visited. Each time the node is visited, the value is updated by

3 $$N(s,a)=\mathop{\sum }\limits_{i=1}^{n}\,{\bf{1}}(s,a,i)$$

The second variable *W* represents the total value of the next state. Every time the node is visited the value is updated to

4 $$W(s,a)=\mathop{\sum }\limits_{i=1}^{n}\,{\bf{1}}(s,a,i){v}_{h}(s,a,L)$$

The variable ${v}_{h}(s,a,L)$ is given by the DNN and represents the value of the actual state at the leaf node L. It is an aggregated function combining the variable $v(s,a)$ and Δ$P(s,a)$. The weights used enable the normalization of both objective.

5 $${v}_{h}(s,a)={w}_{{h}_{1}}v(s,a)+{w}_{{h}_{2}}\Delta P(s,a)$$

The definition given to ${v}_{h}(s,a,L)$ ensures that the solver will link decreased pressure losses to victory and follow the expected trend of the topology optimization. The third variable *Q* represents the mean value of the next state. Once the node is visited, the value is replaced by

6 $$Q(s,a)=\frac{W(s,a)}{N(s,a)}$$

The fourth variable *P* is given by the DNN. It represents the probability of selecting the action associated with the node.

To move from one node to another during the MCTS exploration, the node maximizing *Q* + *U* is selected with U given by:

7 $$U(s,a)\propto \frac{P(s,a)}{1+N(s,a)}$$

A maximum number of nodes **M** is defined to control the exploration. Once **M** is reached, the move is selected based on the maximum value of ${N}^{\tau }$. The value of $\tau$ controls the exploration inside the system and it is chosen $\tau =1$ when exploration is not required anymore.

**Figure 22:**
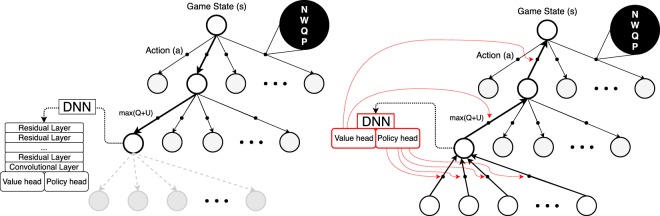
Exploration algorithm in the MCTS. Simulation (left) and backpropagation (right) of the values of interest.

The exploration of the MCTS and node selection is represented in the Fig. 22. The DNN is run at each node with the Game State as input. The output of the DNN gives the values *P* and *v*_*h*_ needed in the MCTS.

### Algorithm structure

**Figure 23:**
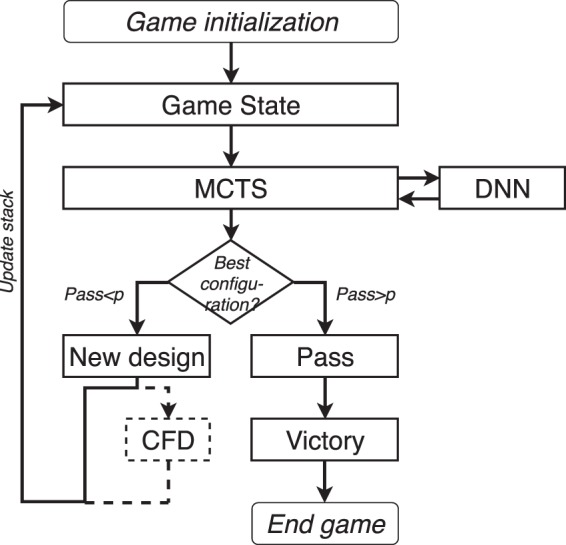
A Game algorithm for FSTO based on ML. There are 2 DNNs, one calibrated for P1 and one calibrated with P2.

**Figure 24:**
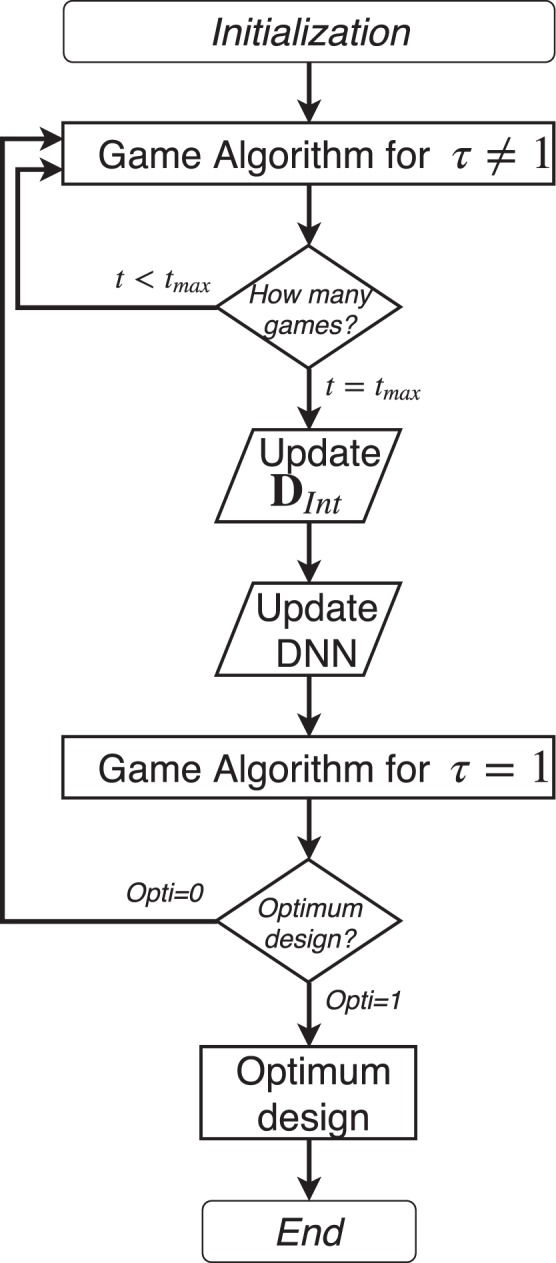
Algorithm of the full process of the optimization, from the initialization up to the optimum design.

To sum up the previous sections, the algorithm run is described in Figs 23 and 24, and it is run for a given period of time. All game states are recorded and once the system is done, the DNN is calibrated with the best *n*_*cal*_ games. The number *n*_*cal*_ is dependent on the test cases. Once the DNN is re-calibrated, the algorithm is run again.

The system stops once a given criteria is met and which yields $opti=1$. For the future test cases it is linked to value of a default value given by the design space. From the moment the default value is reached, the system is let to play until the validation games yield three consecutive identical configurations. A validation game is played after each calibration of the network. This rule can be adapted to the complexity of the problem.

Note that the CFD action only happens if the new configuration has not been visited. If the pressure difference has already been recorded for such design, it will be extracted when calibration is done.

The solver used to compute the difference of Pressure is a modified version of IFISS^21^: Incompressible Flow & Iterative Solver Software. The input of the solver is the final grid output by the system DNN-MCTS, with which it is combined with. There is one grid per player and each grid is tested after being updated if it is a new configuration.

### Calibration and training

The system is self-play. Consequently, calibration and training happens once the system has aggregated enough games. The number of data considered is dependent on the DNN size and the grid size. Smaller test cases will require less data and simple DNN model while larger test cases need mode data and more complicated DNN.

The first random games are added to build the initial dataset **D**_*init*_. As games are played, data are added to build **D**_*Tot*_. The best selected games as corresponding to the criteria of the optimization are input in a new dataset **D**_*Int*_ to calibrate the DNN.

Once a game is finished, the winner is defined by checking the value of the optimization target. For example, if the pressure losses need to be reduced, the best player is defined as the one having the lowest pressure difference and it is confirmed by the modified IFISS^21^. The values obtained for the pressure difference are recorded to build the future training set.

The pressure difference is defined as:

8 $$\Delta P={P}_{outlet}-{P}_{inlet}$$

With

9 $${P}_{{S}_{i}}={\oint }_{{S}_{i}}\,{\bf{P}}\,dA$$

where *S*_*i*_ represents the surface of the inlet or the outlet.

As a matter of information, other objectives can be defined such as increasing temperature exchanged or increasing the diodicity. These objectives would be set up in the training sets and rely on the Fluid solver values. Note that compressible solvers can be used and any type of turbulence models can be added to the RANS simulation.

Utilization of L2 and shuffling of the data is being used while training the data.
